# Methods and research progress in the construction of animal models of osteosarcopenia: a scoping review

**DOI:** 10.3389/fendo.2023.1228937

**Published:** 2023-10-27

**Authors:** Weiwei, Shixuan Wang, Decai Hou, Libin Zhan

**Affiliations:** ^1^ The Second Hospital of Liaoning University of Traditional Chinese Medicine, Shenyang, China; ^2^ Affiliated Hospital of Liaoning University of Traditional Chinese Medicine, Shenyang, China; ^3^ Liaoning University of Traditional Chinese Medicine, Experimental Platform, Shenyang, China

**Keywords:** osteosarcopenia, animal models, aging, review, advantages and disadvantages

## Abstract

**Background:**

Osteosarcopenia(OS) is a significant health concern resulting from the ageing process. Currently, as the population grows older, the prevalence of OS, a disease that entails the synchronous degeneration of muscles and bones, is mounting. This poses a serious threat to the health of the elderly while placing an enormous burden on social care. In order to comprehend the pathological mechanism of OS and develop clinical drugs, it is pertinent to construct an efficient animal model of OS. To investigate the modeling techniques of diverse experimental models of OS and elucidate their respective benefits and drawbacks, with the purpose of furnishing a theoretical foundation to advance experimental research on OS.

**Methods:**

We searched PubMed, Embase database, China Knowledge Network, Wanfang data platform and Vipshop journal platform databases from 2000 through to September 1, 2023. We included animal studies on sarcopenia or osteoporosis or osteosarcopenia or sarcopenia-osteoporosis, modeling methods for osteosarcopenia. Two independently screened study abstracts and full reports and complete data extraction.

**Results:**

Eventually, Of 112, 106 citations screened. 4938 underwent full-text review and 38 met the inclusion criteria. we reviewed and analyzed the literature and categorized the animal models of OS into the following five categories: Aging OS models; Hormonal deficiency model of OS;Chemical injection to induce OS;Disuse OS models and Genetic engineering OS models.

**Conclusion:**

This review outlines animal modeling approaches for OS, providing a comprehensive summary of their advantages and disadvantages. The different models were evaluated and selected based on their respective strengths and weaknesses to enable higher quality research outcomes in various research directions. The most widely used and established approach is considered to be the ageing and chemical injection OS model, which has the advantages of excellent reproducibility and low cost.

**The translational potential of this article:**

To gain a profound comprehension of the pathological mechanism of OS and to devise efficacious clinical treatments, it is imperative to establish a viable laboratory animal model of OS. This article surveys various modeling techniques assessing their benefits, drawbacks and areas of applicability while predominantly employing mice as the primary model animal. Additionally, the evaluation indicators of OS models are briefly described.

## 1 Introduction

The term”OS”is actually used to describe the concomitant manifestation of both sarcopenia (loss of skeletal muscle mass and strength) and osteoporosis (reduced bone mineral density leading to increased risk of fractures). It is a relatively new concept and has gained recognition in recent years due to the recognition of the close relationship between muscle and bone health. While the term “osteosarcopenia” may not be as widely known or referenced as sarcopenia or osteoporosis individually, it is certainly an important syndrome that highlights the detrimental effects of both conditions on musculoskeletal health.

The text describes the relevant elements and factors influencing animal models, as well as illustrating the current problems facing animal models of OS.

The paper outlines that while previous studies have been carried out on this topic, they have not adequately assessed the issue, largely due to small sample sizes or differences in the endpoints under consideration.

OS was first introduced by Neil Binkley in 2009 based on the common pathophysiological features of osteoporosis and sarcopenia (1). OS mainly refers to patients who meet the diagnostic criteria for osteoporosis and also have decreased muscle mass/function. Osteoporosis and sarcopenia are both age-increasing diseases and share many similarities, including high prevalence, high socioeconomic costs, mechanisms of action, and impact on patients’ quality of life (2), both of which can lead to loss of bone mass and muscle mass.

Studies from Australia and China have shown (3) that people with both osteoporosis and sarcopenia have a higher risk of falls and fractures than people with only osteoporosis or sarcopenia. The resulting fractures, particularly hip fractures, were associated with significant morbidity, after a hip fracture, approximately half of patients who could previously walk were unable to move around independently. In addition, 55% of patients over 90 years of age are unable to live independently after a fracture. Frailty is defined as a multidimensional syndrome of loss of reserves (energy, physical, cognitive, health) that leads to vulnerability in older adults. Many older adults suffer from both frailty and osteoporosis and sarcopenia. Locquet M (4)found that after investigating the skeletal muscle health of 288 older adults, the risk of having both osteoporosis was nearly four times higher in patients with sarcopenia compared to non-sarcopenia patients. And another (5) survey of an elderly Chinese population (>80 years) showed that the occurrence of fragility fractures and their complications were significantly associated with OS in 10.4% of men and 15.1% of women.

Therefore, the OS concept promotes holistic thinking about the nature of muscle-bone interactions and offers novel ideas for studying the musculoskeletal system. With the increasing aging of the population, people are paying more attention to OS, and the latest version (2022 edition) of the expert consensus on OS states that “Prevention is more important than treatment” (6). However, research on OS in China is in its early stages, and there is insufficient data from laboratory and clinical studies. This paper aims to examine the relationship between animal models and OS preparation. The authors will sort and summarize the preparation methods of OS animal models, then analyze their respective advantages and disadvantages. This will provide insight into subsequent pathophysiological and therapeutic theoretical studies related to OS.

## 2 Methods

We used the methods for scoping reviews delineated by Arksey and O’Malley, as updated by the Joanna Briggs Institute (7). We report this review according to the PRISMA Extension for Scoping Reviews (8).

### 2.1 Identifification of relevant literature

Two Orthopaedic specialists developed a list of potential keywords. A formal search strategy was then developed by an M.D. and another literature expert, and the literature was searched for concepts including osteoporosis, sarcopenia, and muscular dystrophy.

The search was completed on September 1, 2023 with no language restrictions. We also manually searched the reference lists of included articles.

### 2.2 Search method

①Time frame for the literature search:The time frame for the literature search was 2000-2023.

②Search databases:PubMed, Embase database, China Knowledge Network, Wanfang data platform and Vipshop journal platform.

③Search terms English search term: “osteosarcopenia; Sarcopenia-osteoporosis; Sarcopenia;osteoporosis; Animal models “; Chinese search term: “肌肉减少症;肌少-骨质疏松症; 骨质疏松症; 肌少症; 动物模型”.

### 2.3 Inclusion and exclusion criteria

Criteria for inclusion:

① The article must be about modeling or experimentation with animal models and have final modeling results; ②If it is a duplicate modeling method, choose the modeling method reported in the latest published literature.(For example, two articles from the same experimental team, with exactly the same modeling method, the most recently published article would be chosen); ③ Published in any language (studies that were not published in English were translated using Google Translate. If the article is published in Chinese, it should be at least in a scientific or technical core journal). ④ The model animals should be involved in sarcopenia and osteoporosis.

Literature exclusion criteria:

① Literature that does not match the research(For example, human studies rather than animal model studies;systematic reviews or meta-analyses); ② Duplicate content, ③Articles that cannot be downloaded in full(It’s not a subscription issue, it’s just having the title or comments and not being able to download the full text). ④ No final modeling results.

### 2.4 Study selection

Manual searches were again conducted through the computerized searches described above. When disagreements arose, consensus was reached by two members; if consensus could not be reached, a third member was involved. The selected articles were then reviewed again for full-text relevance by two panelists.

### 2.5 Data extraction

One reviewer screened the data from the selected articles for extraction, and the results were verified by a second reviewer. Data elements extracted included title, author (year), language, modeling method and results. This scoping review focuses on modeling methods and their advantages and disadvantages.Therefore, we did not extract information about the results of the studies, nor did we assess the quality of the included studies.

### 2.6 *Synthesis and* presentation *of* results

We conducted a literature review based on data extracted through descriptive statistics. The benefits and drawbacks of the different modeling techniques are outlined and summarized.

## 3 Results

### 3.1 Results of search

**Figure 1:**
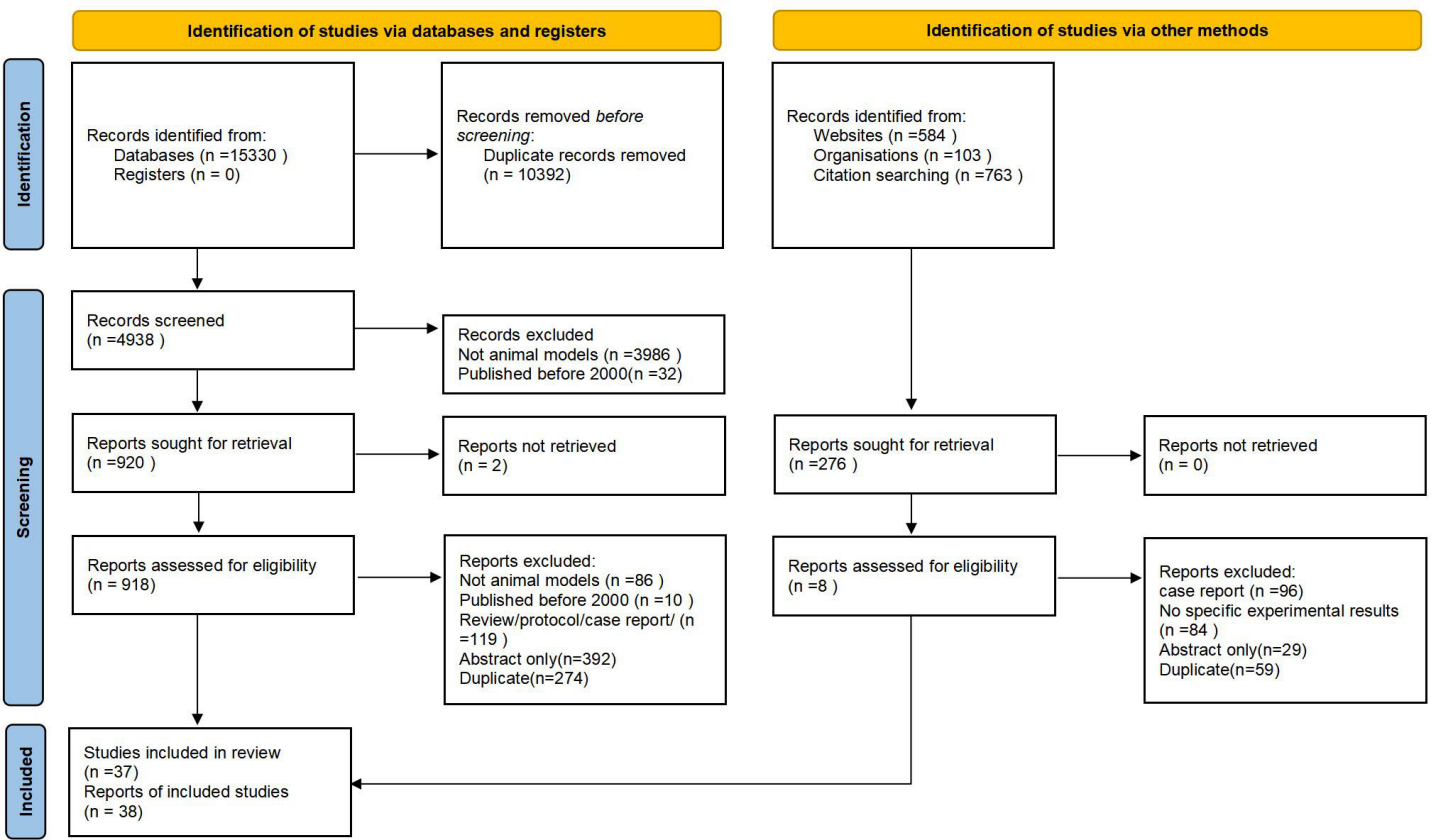
Flow chart of literature screening.

Of these, 10, 392 articles were excluded at the title/abstract review stage (**Figure 1**). Of the 4938 articles selected for full-text review, we excluded 4018. There were 2 full-text articles that we could not locate. After full-text review, we retained 38 articles for data extraction.

### 3.2 Evidentiary features

**Figure 2:**
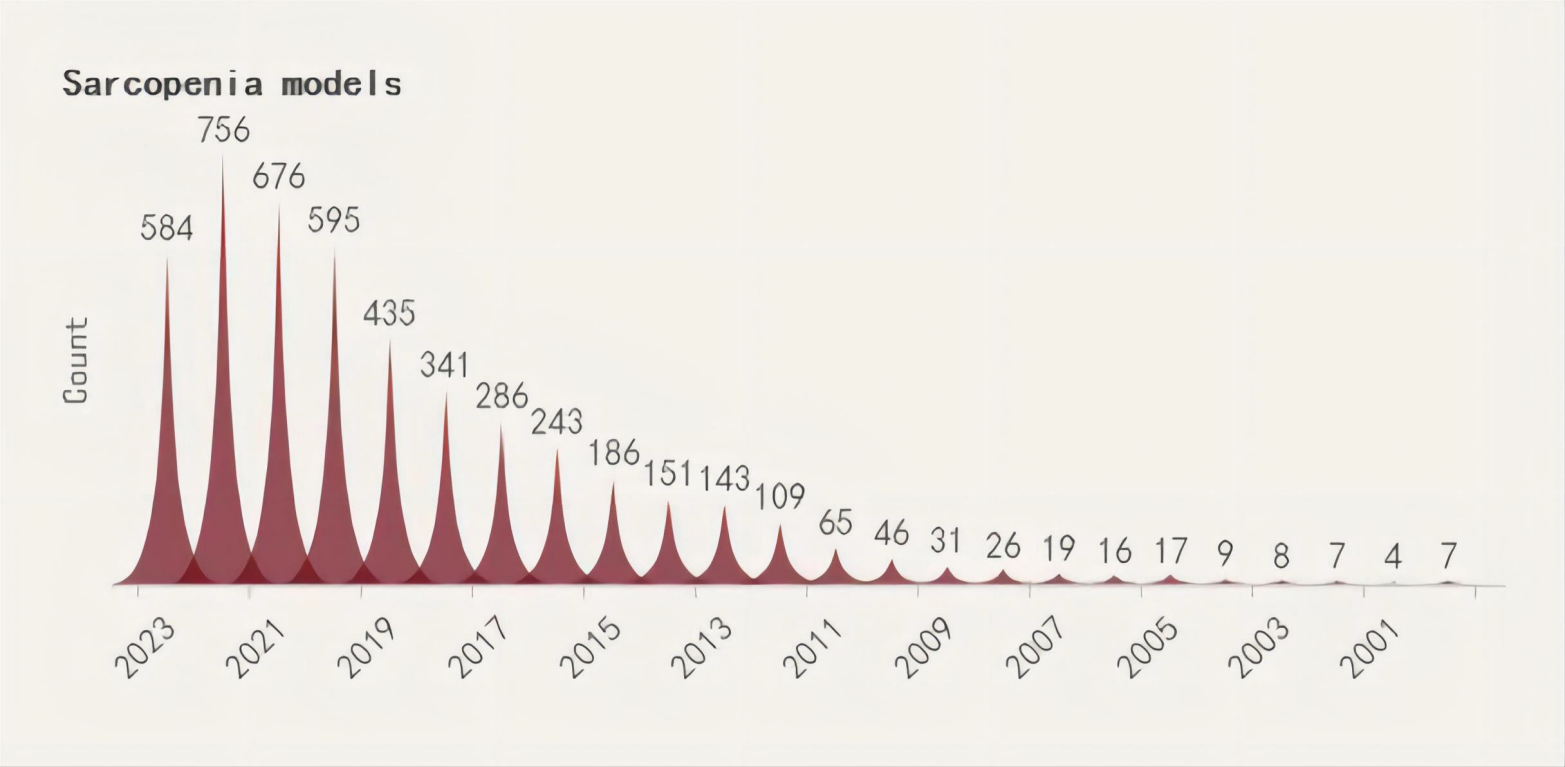
Search query, osteoporosis models (2000-2023).

**Figure 3:**
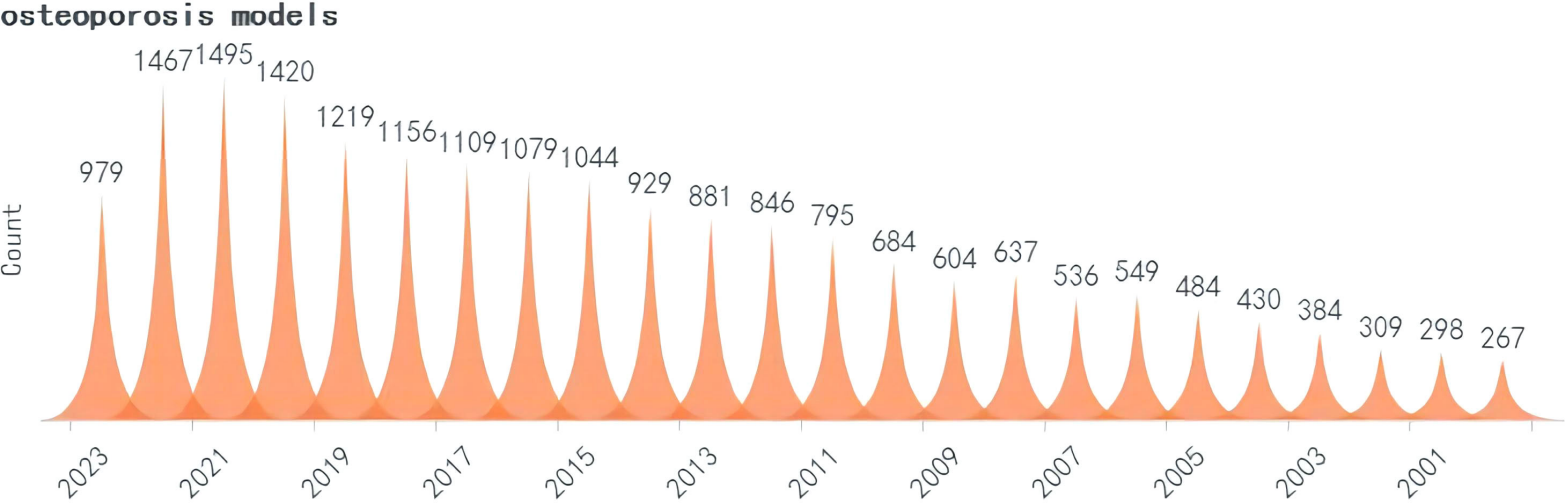
Search query, osteoporosis-sarcopenia models (2000-2023).

**Figure 4:**
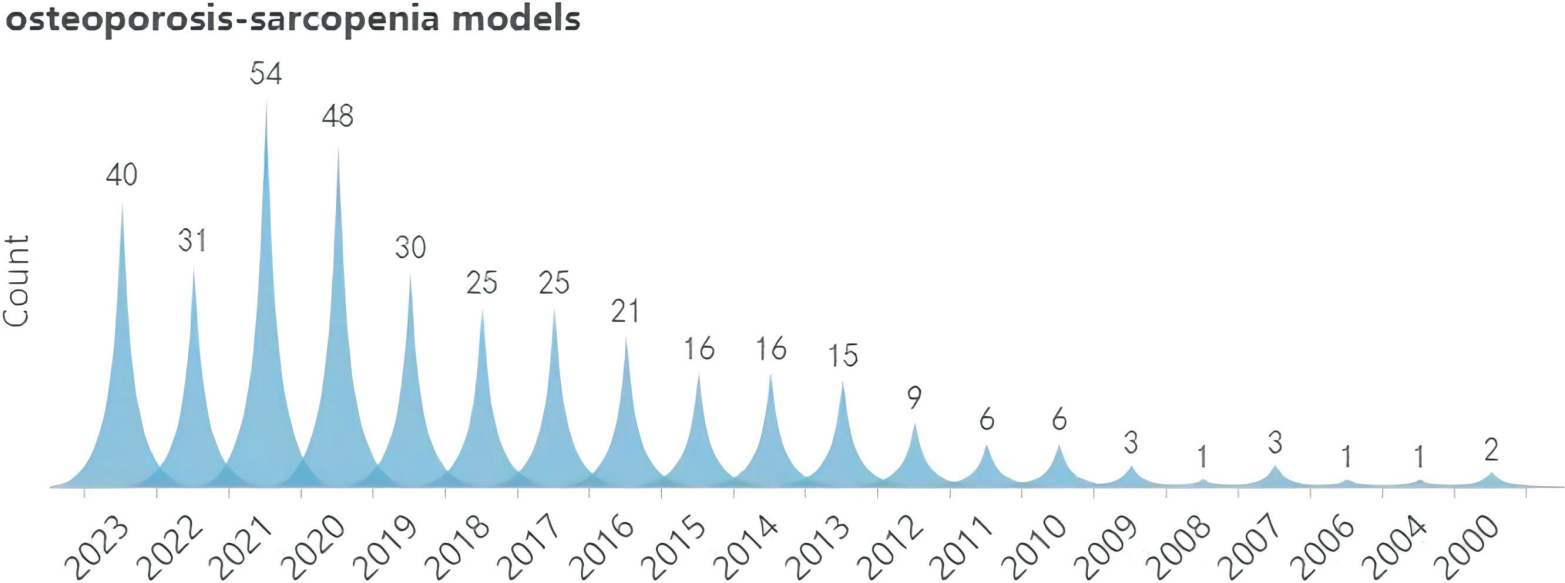
Search query, osteosarcopenia models (2000-2023).

**Figure 5:**
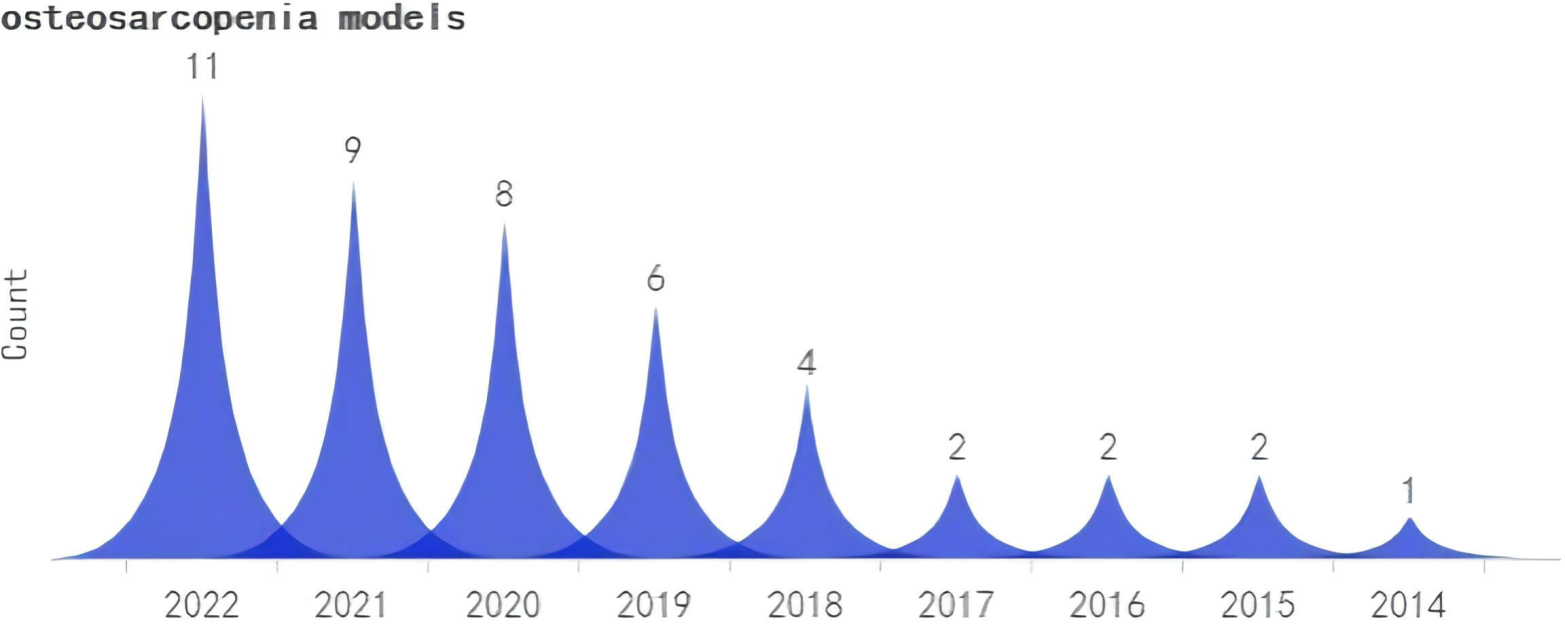
Search query, sarcopenia models (2000-2023).

A search was conducted for all the identified keywords, and their number of relevant publications per year is progressively increasing. Among them, osteoporosis modeling has been researched extensively. The “OS” as a recent terminology has shown an uptick in its frequency of use over the years, albeit relatively less than other related keywords. (**Figures 2**–**5**).

Observational indices differ between models, but typically rely on detecting muscle and bone loss. The table format highlights key aspects of these changes, providing a crucial foundation for OS models (**Table 1**).

**Table 1:** Examples of modeling methods and Indicators evaluated.

Author	Modelling method	Indicators evaluated	Year	Literature
***Kondratov RV***	Aging	Developmental retardation: growth retardation around 16-18 weeks; reduced muscle mass and fat (mean abdominal mass 1.5g at 40 weeks of age); reduced mass of vital organs (vital organs such as spleen, testes, heart, and lungs in 40-week-old animals); and altered peripheral blood in the KO mouse model (the sum of neutrophils and monocytes at 40 weeks of age was twice as high as in the control group)	2006	9
***Hosokawa M***	Aging	Viscera show higher oxidative status; mitochondrial dysfunction and high oxidative status (reduced Cu, Zn-SOD activity in mitochondria)	2002	10
***Azuma K***	Aging	Elevated levels of oxidative stress and mitochondrial dysfunction in tissues, muscle loss, osteoporosis	2018	11
***Borbélyová V***	Aging	Bone mineral density (13% lower) and bone mineral content (15% lower); led to increased adiposity (26%); lower body weight (44%)	2021	12
***Alyodawi K***	Ercc1Δ/- premature aging model	40%-60% reduction in hindlimb muscle mass compared to normal muscle mass	2019	13
***Iemura S***	Excision of mouse testes	Mouse flounder muscle atrophy	2020	14
***Borbélyová V***	Gonadectomized mice	Bone mineral density (13% reduction) and bone mineral content (15% reduction)	2021	15
***Gomes RM***	Ovariectomised mice	Decreased bone density; decreased muscle mass	2018	16
***Ma Jiangtao***	Debridement combined with intraperitoneal dexamethasone injection	Rat forelimb grip strength decreased significantly; bone density decreased; bone volume of distal femur decreased significantly, trabecular thinning, trabecular number and thickness decreased significantly, trabecular separation increased significantly	2022	17
***Gasparini SJ***	Corticosterone delivery	Increased fat content; decreased serum calcein levels; decreased bone density	2016	19
***Pal S***	Injection of methylprednisolone	Significant reduction in gastrocnemius cross-sectional area; decreased bone mass	2020	20
***Mito T***	Hormone Model	Decreased muscle mass of tibialis anterior muscle; decreased number of bone trabeculae	2015	21
***Papadopoulou SK***	Suspension	Muscle atrophy, loss of bone mass.	2021	38
***Du F***	uspended Matching Plaster	Decrease in cross-sectional area of rat toe extensors; transformation of type IIB fibers into type I and type IIA fibers	2011	39
***Palus S***	plaster immobilization	Loss of bone mass, muscle atrophy	2017	40
***Brent MB***	Botulinum toxin	Braking-induced BV/TV was completely offset by reduced muscle mass (-63%), reduced trabecular BV/IV (-28%), reduced Tb.Th (-11%), reduced cortical thickness of the diaphysis (-10%), and reduced bone strength of the distal femoral epiphysis (-27%)	2018	41
***Sataranatarajan K***	genetic engineering	Loss of muscle mass and strength, loss of skeletal muscle innervation, and increased skeletal muscle proteolysis	2020	42
***Mito T***	genetic engineering	Bone density, muscle atrophy of the quadriceps, and ultimately osteoporosis and sarcopenia were observed in all mice	2013	43
***Hey HWD***	Genetic Engineering	Loss of disc height; loss of paraspinal muscle content Upregulation of FGF-21 negatively regulates bone homeostasis	2022	44

### 3.3 Aging model

Aging is the most common and currently the leading clinical cause of OS. In this section, we provide an overview of several mouse models of aging linked to OS, and assess their respective merits and demerits (**Table 2**).

**Table 2:** Advantages and disadvantages of aging models.

Models	Modeling method	Advantages	Disadvantages	Suitable research direction	Literature
Aging models	Natural aging rat models	Mimics human aging process, no drug residue in the body, more natural	Higher time costs, high feeding costs,	Aging-related diseases	
	Disruption of circadian rhythms observed in specific strains of Bmal1 KO mice	Accelerates aging, saves costs, and can exhibit late aging characteristics	Experimental animals are expensive	Aging-related diseases	9
	SAMP mouse strains, accelerated aging model	Accelerates aging, saves costs, and can exhibit late aging characteristics	Average survival rate	Oxidative stress levels and direction of mitochondrial dysfunction, age-related osteoporosis and degenerative temporomandibular joint disease	10-11
	OXYS rats	Preparation of an OS model along with other aging features such as arterial hypertension, sarcopenia and neurodegenerative changes in the brain	Species are not easy to obtain	The study of aging mechanisms and the pathogenesis of age-related diseases can also be used to objectively evaluate new therapeutic and preventive approaches	12
	Ercc1Δ/- premature aging mouse model	Accelerated aging while having aging characteristics	Loss of DNA repair activity and expensive animals	For genetic mutations causing aging	13

First, the natural aging rat model has a lifespan of about 2.5-3 years, and old age is usually after 20 months, which is equivalent to after 55 years of age in humans. It is generally recognized that OS may be observed after 18 months of age. However, due to the high time cost of culturing naturally aging rats, further studies and reports are needed for this disease.

In studies of artificially intervened, pro-aging or premature aging models, Kondratov (9) observed specific strains of Bmal1 knockout mice (Bmal1 KO) by disrupting circadian rhythms and found that Bmal1 KO mice had a shortened lifespan and exhibited features of late aging with significant changes in skeletal structure as well as various other lesions. These mice also exhibited significant skeletal muscle weakness and structural pathological changes with altered myofilament structure and abnormal mitochondrial volume and function. One of the earliest models of accelerated senescence was the inbred strain of the accelerated senescence propensity (SAMP) mouse (10), which is now hypothesized to be due to elevated levels of oxidative stress in tissues and mitochondrial dysfunction, leading to senescence. Certain SAMP strains have been found to have musculoskeletal defects, mainly SAMP6 and SAMP3, which exhibit senile osteoporosis and degenerative temporomandibular joint disease, respectively, at approximately 3 months of age (11). Borbélyová (12) selected galactose-sensitive Wistar rats for inbreeding to create genetic models of accelerated aging and related diseases (OXYS rats), and currently their team has the 103rd generation of OXYS rats, which, in addition to cataracts, are characterized by osteoporosis, arterial hypertension, sarcopenia and neurodegenerative changes in the brain. Thus, this rat model can be used not only to study the mechanisms of aging and the pathogenesis of age-related diseases, but also to objectively evaluate new therapeutic and preventive approaches.

Human progeria and progeroid syndromes are genetic disorders and accelerated aging due to loss of DNA repair activity is one of the more studied bases of progeria models.Alyodawi K (13)found that Ercc1Δ/- progeria mouse model is similar to naturally aging rodents and has several significant features at the same time (e.g. sarcopenia, osteoporosis, degeneration of liver and kidney function, neuropathy, etc). The mechanism is due to mutations in the Ercc1 subunit of the XPF-ERCC1 endonuclease they carry. XPF-ERCC1 nucleases are involved in nucleotide excision repair, interstrand crosslink repair and double-strand break repair, and XPF-ERCC1 plays an important role in many types of DNA repair. Thus, patients with XPF mutations exhibit significant signs of accelerated aging. There is evidence that complete deletion of Ercc1 or XPF results in dramatically accelerated aging and a significantly shorter lifespan (only 1 month) in mice. However, in Ercc1-/Δ mice, aging is accelerated when XPF/Ercc1 is expressed at only 5%-10% of normal levels.

Analysis of different models of aging, including a mouse model of human progeria, suggests that treatment of naturally aging mice with certain compounds that extend healthy lifespan and/or longevity improves pathology in models of accelerated aging.Pathways such as DNA damage, oxidative stress, and stem cell dysfunction all play a role in accelerating and driving the musculoskeletal aging process. These findings clearly demonstrate the utility of therapeutic approaches to slow musculoskeletal aging. By using conditional transgenic models that selectively accelerate aging by targeting specific genetic defects, such as loss of DNA repair and reduced nuclear structure or chromosome segregation, it is possible to assess the non-cell-autonomous effects of specific tissues (e.g. bone or skeletal muscle) on the aging of other tissues.

A single mouse model has not yet emerged that fully represents all aspects of biological aging. However, detailed transcriptomic, proteomic, and metabolomic analyses suggest that among the many models of accelerated aging, the Ercc1-/Δ mouse model best reflects natural aging, including aging of the musculoskeletal system. The use of these whole-body accelerated aging models, combined with tissue-targeted aging mouse models, will not only accelerate the understanding of the mechanisms driving musculoskeletal aging, but will also facilitate the development of drugs that can prolong musculoskeletal health.

### 3.4 A hormone-deficient model of OS

It is well known that androgen deficiency leads to osteoporosis and sarcopenia. Myokine, a humoral factor secreted by skeletal muscle, has received attention as a key factor in the interaction with muscle and bone (**Table 3**). Orchiectomy, the surgical removal of testes, is performed on male rodents to create a model of hypogonadism-related sarcopenia and osteoporosis. This model helps researchers understand the role of androgens, such as testosterone, in the regulation of muscle and bone health and evaluate the efficacy of androgen replacement therapies. Iemura S (14) show that androgen deficiency increases the expression and secretion of Dickkopf (Dkk) 2, an inhibitor of Wnt/β-linker signaling, which is essential for maintaining muscle and bone function, in the muscles of mice. The research team initially demonstrated that Dkk2 plays a role in muscle and bone alterations in androgen-deficient mice. *In vivo* experiments revealed a negative correlation between serum Dkk2 levels and bone mineral density in mouse tibial trabeculae. *In vitro* experiments have shown that testosterone suppresses Dkk2 mRNA levels in rat muscle cells. It is therefore hypothesized that the myokine Dkk2, which connects muscle and bone, may be linked to muscle atrophy and bone loss resulting from androgen deficiency.

**Table 3:** Advantages and disadvantages of hormone deficiency models.

Models	Modeling method	Advantages	Disadvantages	Suitable research direction	Literature
Hormone deficiency models	Excision of mouse testes	Easy and reproducible experiments	Androgen deficiency-induced OS	Osteoporosis and sarcopenia in men - osteoporosis	14
	Gonadectomy	Long-term hypogonadism models have research advantages over short-term hypogonadism models	Id.	Id.	15
	OVX rats	The most common effects of postmenopause are related to the malignant triad, including loss of bone and muscle mass, and increased physical obesity	Restricted to postmenopausal women	Postmenopausal women	16
	OVX combined with dexamethasone injection	short modeling cycle, stable model, high operability and low cost.	Restricted to postmenopausal women	Postmenopausal women	17

Borbélyová V (15) conducted a study in which they randomized male rats into control and gonadectomy groups. The male rats underwent gonadectomy on the 47th day of life and were then raised for up to 30 months. The results of the study showed that prolonged androgen deficiency resulted in reduced muscle mass compared to the control group. Androgen deficiency lasting more than two years resulted in severe loss in muscle mass and slower fat mass gain in older rats. This long-term hypogonadism model can simulate male patients with OS and offers a greater research advantage than the short-term hypogonadism experimental model currently used in young animals.

Osteoporosis in postmenopausal women is currently a widely researched issue. However, often only the reduction in bone mass following menopause is acknowledged, with little attention given to the loss of muscle tissue. Gomes RM (16) considered the ovariectomised (OVX) rat as an animal model to simulate the postmenopausal situation. The most common postmenopausal effects are associated with the malignant triad, including loss of bone and muscle mass and increased body fatness. The research team randomized female Wistar rats into non-ovariectomized rats (Sham), OVX rats, 17β-estradiol (HR)-treated OVX group, and OVX-trained group (TR). At 70 days of age, the OVX group underwent bilateral ovariectomy to successfully establish an OS model in the rats. The study found that estrogen deficiency plays a key role in bone and muscle loss. They concluded that bone density, muscle strength and muscle mass can be improved by strength training to prevent or ameliorate muscle loss and bone loss due to reduced ovarian hormone levels. A recent study in China reported that Jiangtao Ma (17) applied OVX combined with intraperitoneal injection of dexamethasone to establish a rat model of OS. After three months, BMD, grip strength, and muscle content were measured, and the OS rat model was successfully prepared. The researcher concluded that this composite modeling method offers several advantages, including the short modeling period, stable model, operational feasibility, and low cost. And the intervention was carried out by Chinese herbal bone strengthening and pain relief formula, which was considered to have a certain therapeutic effect on OS (18).

### 3.5 Induction of OS by chemical drug injection method

Chemical injections are often used in the laboratory to establish animal models, with the most common chemical being glucocorticoids (GC), and it has been found that overdose of GC can induce bone loss, muscle atrophy, and decreased immunity, so many researchers have used this as a starting point to employ a hormone-induced OS model (**Table 4**). Gasparini SJ (19) conducted a study to compare the effects of two methods of corticosterone (CS) delivery in a rodent model. One was the delivery of CS (at concentrations of 25-100μg CS/mL) via drinking water and the other was the delivery of 1.5 mg CS via surgically implanted slow-release pellets for a total of 4 weeks of experimentation. It was found that both methods induced typical adverse outcomes such as fat accumulation, insulin resistance, muscle loss and osteoporosis. The team concluded that this method is safe, inexpensive, easy to regulate, and avoids stressful handling of the animals. And in a study by PAL (20), they selected male SD rats and administered 5 mg/kg of methylprednisolone (MP) daily via subcutaneous injection for 4 weeks. At the end of the treatment, they examined bone density, muscle content and micro-crt and successfully modeled osteoporosis.Mito T (21) used animal models of diabetes mellitus (DM) to track metabolic changes in skeletal muscle and bone to provide important insights into the management of diabetic complications. They used streptozotocin (STZ) to induce a type 1 DM mouse model and then applied dual-energy X-ray absorption (DXA) to determine their fat mass, skeletal muscle mass (lean body mass), bone mineral density, and bone mineral content. In the DM model, STZ administration resulted in elevated blood glucose levels, increased water and food intake, and weight loss. Anterior tibial (TA) muscle weights were measured after 30 days and tibial micro-structure was quantified by micro-computed tomography imaging in DM mice. The TA muscle weights were significantly lower in DM mice than in control mice. Furthermore, the trabecular bone volume fraction, trabecular thickness, trabecular number, and cortical thickness were substantially reduced in mice with diabetes mellitus. The method employed successfully established a mouse model of osteoporosis, presenting a novel approach to investigating diabetic complications.

**Table 4:** Advantages and disadvantages of chemical injections to induce OS.

Models	Modeling method	Advantages	Disadvantages	Suitable research direction	Literature
Chemical injections to induce OS	Implantation of extended-release pellets containing 1.5 mg CS for a total of 4 weeks	Method is safe, inexpensive, and avoids repetitive operations	Higher operational requirements and no other aging process characteristics	Can be used for hormone-induced OS	19
	Subcutaneous injection of 5 mg/kg of MP for 4 weeks	Safe, inexpensive, easy to adjust and low cost method	No other aging process characteristics	Id.	20
	STZ induced type 1 DM mouse model	First diabetes-related OS	Smaller research areas	New ideas for the study of diabetic complications	21

### 3.6 The disuse OS model

Disuse muscle hypo-osteoporosis is more common in clinical settings such as prolonged bed rest (22), immobilization after motor neuron disease (23), treatment of fractures with splints or casts, or exposure to microgravity during space flight (24). In particular, such models can be better prepared for patients with OS who are bedridden for long periods of time after fracture surgery, hemiplegia, or cerebrovascular disease, when lack of stressful stimulation and weightless activities lead to bone loss and muscle atrophy (**Table 5**). Most studies (25–31) have found that exercise improves muscle mass, strength, and function, and therefore may be protective against sarcopenia by increasing muscle mass and strength and improving mobility, while less active individuals have an increased risk of developing sarcopenia or increasing its severity.

**Table 5:** Advantages and disadvantages of the disuse OS model.

Models	Modeling method	Advantages	Disadvantages	Suitable research direction	Literature
Disuse OS models	TS+Im for OS model preparation	Method is safe, inexpensive, easy to adjust, low cost, and can mimic post-operative fracture patients	Prone to other complications, such as blood clots and pressure sores	The principle is thought to promote muscle atrophy by disrupting a variety of cellular processes, such as the induction of oxidative imbalances, mitochondrial dysfunction	39
	Plaster fixation	More effective for muscle loss	Regular observation Prevent limb necrosis	Disuse muscle hypo-osteoporosis	40
	4IU of BTX was injected into the right hind limb muscle tissue of 12-14 week old female Wistar rats to prepare an OS model	Safe, inexpensive, easy to adjust and low cost method	Inconsistent with human disease course	For muscle reduction direction helps more	41

Despite heterogeneity among studies, most studies suggest that weight-bearing and resistance exercise are most effective in the prevention and treatment of osteoporosis in older adults (32–36). The relationship between osteoporosis and sarcopenia makes sense in the context of the bone-muscle subunit. Both tissues are derived from a common type of mesenchymal projection stem cells. Muscle cells secrete bone-regulating cytokines, whereas bone cells secrete Insulin-like growth factor 1(IGF-1), which has potential muscle-stimulating properties (37).

Suspension of the hind limb by tail suspension or sling is the most commonly used method to induce a disused model (38). Tail suspension fixation is an effective model of hindlimb disuse. Unloading or immobilization models are used to study the effects of disuse on muscle and bone. This can be achieved through methods such as tail suspension, hindlimb unloading, or casting in rodents. These models assist researchers in exploring the mechanisms of disuse-induced muscle atrophy and bone loss, while also evaluating potential interventions to mitigate these effects.du F (39). Prepared an OS model by tail suspension (TS) and one hind limb (Im) fixed by plaster-casting. Their team suspended female Sprague-Dawley rats by their tails for 6 weeks. Significant changes in muscle atrophy were found at 4 weeks, and after 6 weeks the muscle fiber type of the rats was measured and a shift from oxidative to glycolytic metabolism was found in the finger extensor muscle fibers. The rationale for the suspension OS model is thought to promote muscle atrophy by disrupting multiple cellular processes, such as induction of oxidative imbalance, mitochondrial dysfunction, intercellular interactions, and abnormal protein synthesis/degradation.

Palus S (40) concluded that plaster fixation is the most commonly used model to study muscle atrophy because it simulates plaster fixation after fracture, wraps the leg with a plaster bandage or spiral wire, and allows for prolonged immobilization. Loss of bone mass is observed along with muscle atrophy, which is consistent with what is observed clinically, and its believed that this model can be used to assess muscle loss. Botulinum toxin (BTX) is now also used in the preparation of disuse models. Brent MB (41) conducted a study to establish a model of disuse OS by injecting 4 IU of BTX into the muscle tissue of the right hind limb of 12-14 week old female Wistar rats. After 6 weeks of intervention, the research team looked at the preventive or ameliorative effects of intermittent parathyroid hormone (PTH) and growth hormone (GH), alone or in combination, on wasting bone loss and muscle loss in rats. The results of the study found that PTH completely counteracted fixation-induced loss of skeletal microarchitecture, density, and distal femoral bone strength.GH increased muscle mass but did not prevent fixation-induced bone loss. However, the combination of PTH and GH increased distal femoral bone strength, skeletal microarchitecture, density, and bone density throughout the femur, as well as increasing muscle mass. Overall, the combination of PTH and GH was more effective in preventing abolition-induced bone deterioration than was its application alone. In addition, GH alone or in combination with PTH attenuated wastage-induced loss of muscle mass. Thus, the combination of PTH and GH is more effective than its application alone.

### 3.7 Genetic engineering OS model

Genetic engineering, also known as gene splicing technology and DNA recombination technology, is based on the theory of molecular genetics and the modern methods of molecular biology and microbiology, to construct *in vitro* hybrid DNA molecules from different sources according to a predesigned blueprint and then introduce them into living cells to change the original genetic characteristics of organisms, obtain new varieties and produce new products. The purpose of genetic engineering is to modify the genetic characteristics of organisms, to obtain new varieties, and to produce new products. Depending on the technical approach, genetically engineered animal models can be classified as transgenic animal models, knockout/knock-in animal models, and genetically engineered disease animal models (**Table 6**). Sataranatarajan K (42) suggested that aged wild-type mice usually do not show the OS phenotype until late in life (26 months or longer). For this reason, the team used genetic engineering to prepare Sod1-/- mouse models that exhibit many of the pathological features of aging at approximately 8 months of age, such as loss of muscle mass and strength, loss of skeletal muscle innervation, and increased skeletal muscle protein catabolism, which can be used to study aging-related sarcopenia and osteoporosis. The rationale is that mitochondrial dysfunction and increased hydroperoxide production induce skeletal muscle atrophy. Mito T (21) suggested that the phenotypic profiles associated with aging and mitochondrial disease appear to overlap. The research team conducted a comparative study on aging mice using a mitochondrial disease mouse model (mtDNA-deficient mitochondrial mitotic mice). Their findings revealed that osteoporosis and sarcopenia were observed in all mice upon comparing bone density and quadriceps muscle atrophy. This confirms that mtDNA mice exhibit phenotypes similar to those observed in the aging population (sarcopenia, osteoporosis).Clinical OS does not only occur in the elderly population. Duchenne muscular dystrophy (DMD) is the most common form of muscular dystrophy in children. In addition to skeletal muscle, DMD also has significant effects on the skeleton. methods for the preparation of animal models of DMD are becoming increasingly sophisticated, but the pathogenesis of skeletal abnormalities in DMD remains unknown. Recently, Hey HWD (43) identified a novel bone regulatory cytokine, fibroblast growth factor-21 (FGF-21), which is significantly up regulated in skeletal muscle in animal models of DMD. The hypothesis that muscle-derived FGF-21 negatively affects bone homeostasis in DMD was proposed and experimentally investigated by using dystrophin/pro-ovalbumin double knockout (dKO) mice. The final study showed that dystrophic skeletal muscle expresses and secretes significant levels of FGF-21, which negatively regulates bone homeostasis and is an important pathological factor in the development of bone abnormalities in DMD. The study highlights the importance of muscle/skeletal crosstalk via muscle-derived factors (myokines) in the pathogenesis of bone abnormalities in DMD.

**Table 6:** Advantages and disadvantages of the Genetic engineering OS models.

Models	Modeling method	Advantages	Disadvantages	Suitable research direction	Literature
Genetic engineering OS models	Preparation of Sod1-/- mouse models	Short time and easy access (approximately 8 months of age)	High cost	Skeletal muscle atrophy is induced by mitochondrial dysfunction and an increase in hydroperoxide production.	42
	mtDNA-deficient mitochondrial mitotic mice	Mimicking aging mice	Genetic defects, high costs	Mitochondrial mitotic defects	43
	Experimental study of myotonic dystrophy protein/pro-ovalbumin dKO mice	For DMD research		Dystrophic skeletal muscle expression, Duchenne muscular dystrophy in children	44

## 4 Discussion

### 4.1 Selection of model animals

A good disease model should have the following characteristics: ① able to reproduce the human disease to be studied; ② animals can repeatedly produce the disease, which can be standardized; ③ complete background information of the animals, the life cycle to meet the needs of the experiment; ④ the animals should be inexpensive, adequate sources, easy to transport; ⑤ as far as possible, the use of small animals (44).

The most commonly used model animals in medical research are rodents, and the mouse genome is similar to that of humans and is easy to manipulate (45).

OS becomes more common with age and is associated with muscle weakness, disability, falls and fractures, and increased morbidity and mortality (46–50). Therefore, most of the old rats or prematurely aged rats are used for model preparation, and 3-6 months old rats are currently considered by researchers as the best period to observe bone microstructural changes after de-ovulation, and are a more ideal experimental animal model to study postmenopausal osteoporosis (51). However, some studies have shown that rhesus monkeys are the best model for human sarcopenia. Unlike rodents, where significant muscle mass loss occurs quickly later in life (52), the kinetics of sarcopenia in rhesus monkeys match those of humans, with onset in middle age and gradual reduction thereafter. The reduction in muscle fiber cross-sectional area significantly contributes to muscle mass loss and an age-dependent increase in muscle fibers with mitochondrial enzyme abnormalities due to mitochondrial DNA deletion mutations has been observed (45, 53). In addition, skeletal muscle accounts for a greater proportion of total body weight in primates compared to rodents and is a huge consumer of energy expenditure.

### 4.2 Indicators for the evaluation of OS

Most of the evaluation indices of animal models of OS are analyzed and evaluated from both muscle and bone aspects together, whereas animal models of sarcopenia are tested at muscle mass, muscle strength and cellular level. Muscle mass can be responded by radiography (CT, MRI), electromyography; muscle strength is most commonly tested by the test of limb grip strength, and at the molecular biology level, the general test of myogenic cell proliferation or myotubular formation speed methods.

The assessment of osteoporosis in skeletal tissue can be accomplished through multiple approaches. Measuring bone mineral density is a direct and easily observable method to verify if the index is in line with osteoporosis diagnosis. Alternatively, Micro-CT analysis can be conducted to further examine bone morphology. Lastly, the application of HE staining can facilitate an observation of the condition of bone cells and the microstructure of bone trabeculae following the material extraction.

### 4.3The most commonly used and proven models

Combining the above methods of preparing models, the author believes that the most commonly used and proven methods in preparing OS are the aging model and the chemical drug injection model.

The following is a more detailed analysis:

①Pathological Characteristics Simulation: Aging Model and Chemical Injection Model are two effective methods for simulating pathological features. The Aging Model mimics the natural ageing process in humans and accurately simulates the pathological features of sarcopenia and osteoporosis, resulting in study findings that are consistent with real disease. The Chemical Injection Model, on the other hand, uses specific drugs such as hormone inhibitors to accurately simulate the features of muscle loss and osteoporosis in model animals, leading to similar pathological manifestations.

②Controllability : Aging Models: In aging models, the pathogenesis of the model can be controlled by selecting experimental animals of varying ages. By regulating the age of the experimental animals, one can observe and analyze the changes of OS at different stages, thereby exploring the pathogenesis at varying points in time. Chemical injection model: The degree of sarcopenia and osteoporosis can be precisely controlled in model animals by regulating the dosage and frequency of drug injection. Researchers are able to select the appropriate dose and adjust the rate of disease progression by controlling the duration of drug administration.

③Reproducibility: The aging model displays natural age-related changes in the senescence model, allowing for good reproducibility when using the same animal lines and age ranges in various laboratories and research conditions. This facilitates comparison and validation of research outcomes among different laboratories. The reproducibility of chemical drug injection modeling can be improved by controlling the type of drug, dosage, and injection method. The identical pharmaceutical and injection protocol may yield comparable pathological influences in various laboratories, thereby enabling dependable assessment and authentication of research outcomes.

④Cost-effectiveness: Aging Model: Compared to some other models of OS, the aging model necessitates neither complex gene editing nor special feeding conditions, resulting in lower research costs. Chemical Injection Model: This model is relatively simple, as it only requires the selection of appropriate drugs and dosages, and its cost-effectiveness makes it suitable for research purposes.

By combining the benefits of the aging model and the chemical drug injection model, a more detailed comprehension of the pathogenesis and influencing factors of OS could be attained. Selecting the suitable method from these two models, depending on the specific experimental requirements and research goals, can enhance the dependability of the outcomes and facilitate further exploration of the disease.

## 5 Summary

Osteoporosis and sarcopenia are two diseases that share many similarities, including high prevalence, high socioeconomic costs, mechanisms of action, and critical impact on patients’ quality of life (54). Both are capable of causing loss of bone mass and muscle mass and are age-related. In addition, sarcopenic obesity observed in the elderly may increase the risk of cardiometabolic disease, disability and death and accelerate the decline of somatic function (55). Obesity, sarcopenia and osteoporosis may coexist and such patients are prone to more severe health problems compared to patients with one of these conditions (56).

The core of the pathological mechanism of OS is the degeneration of muscle and bone, and more and more studies are now confirming the endocrine relationship between muscle and bone, such as skeletal muscle secretion of myostatin, irisin and other actin factors, which not only regulate skeletal muscle growth but also affect bone metabolism to some extent (57). This is the theoretical basis on which the modeling approach can successfully construct an OS model. More and more scholars pay attention to OS, and contribute to the further development of clinical medicine by preparing models to better explore the relationship between the two. However, most of the animal models at the present stage focus on a certain etiology or a certain stage of pathological changes of OS, and cannot fully reflect the pathological process of OS in humans. In addition, different modeling approaches have their own advantages and disadvantages, and the most suitable modeling approach should be selected when designing the experiments according to the different etiologies and pathological stages of OS. Future research will seek to address the limitations in each modeling approach. This will enable the design of OS models to adhere to key principles such as similarity, reliability, reproducibility, applicability, controllability, ease of use and economy. Such advancements will undoubtedly contribute to the in-depth investigation of OS, and ultimately improve the development of effective clinical drugs.

In conclusion, the use of different animal models has significantly enriched our comprehension of sarcopenia and osteoporosis. Every model has its own pros and cons, and selecting an appropriate model relies on the particular research question and hypothesis under examination. By utilising animal models, researchers can further investigate the intricate interplay between muscle and bone health and advance new therapeutic approaches to prevent and treat sarcopenia and osteoporosis.

## Author contributions

WW had full access to all the data in the study and takes responsibility for the integrity of the data and the accuracy of the data analysis. WW and SW conceived the study, developed the research protocol, and selected the studies to be reviewed. SW and DH developed the search strategy and conducted the searches. WW and LZ extracted the study data. WW drafted the manuscript. All authors contributed to the article and approved the submitted version.
